# Evaluating the Reliability and Consistency of Treadmill Gait Analysis Using an RGB-D Camera: Effects of Assistance and No Assistance

**DOI:** 10.3390/s25020451

**Published:** 2025-01-14

**Authors:** Yuichiro Hosoi, Takahiko Sato, Akinori Nagano

**Affiliations:** 1 Graduate School of Sport and Health Science, Ritsumeikan University, Kusatsu 525-8577, Shiga, Japan; 2 Department of Rehabilitation Medicine, Keio University School of Medicine, Shinjuku-ku 160-8582, Tokyo, Japan; 3 Faculty of Rehabilitation, Biwako Professional University of Rehabilitation, Higashiomi 527-0021, Shiga, Japan; 4 Institute of Advanced Research for Sport and Health Science, Ritsumeikan University, Kusatsu 525-8577, Shiga, Japan; 5 College of Sport and Health Science, Ritsumeikan University, Kusatsu 525-8577, Shiga, Japan

**Keywords:** markerless, gait analysis, reliability, RGB-D cameras

## Abstract

This study aimed to assess the intraday reliability of markerless gait analysis using an RGB-D camera versus a traditional three-dimensional motion analysis (3DMA) system with and without a simulated walking assistant. Gait assessments were conducted on 20 healthy adults walking on a treadmill with a focus on spatiotemporal parameters gathered using the RGB-D camera and 3DMA system. The intraday reliability of the RGB-D camera was evaluated using intraclass correlation coefficients (ICC 1, 1), while its consistency with the 3DMA system was determined using ICC (2, 1). The results demonstrated that the RGB-D camera provided high intraday reliability and showed strong consistency with 3DMA measurements regardless of the presence of an assistant. The Bland–Atman analysis indicated no significant systematic bias, with the minimum detectable change remaining within acceptable clinical ranges. These findings highlight the potential of the RGB-D camera for reliable markerless gait analysis in clinical environments in which walking assistance may be needed, thereby expanding its applicability in patients with various impairment degrees. Future research should validate these results in patient populations and explore their utility for measuring kinematic parameters.

## 1. Introduction

In the field of rehabilitation, gait analysis plays an important role in identifying the cause of deviations from normal gait, selecting and implementing appropriate intervention methods, and evaluating their effects [1,2,3]. Previous reviews have reported that gait analysis contributes to the clinical diagnosis and treatment selection decisions of clinicians and physiotherapists [1,2,4,5].

The primary gait analysis method used in clinical settings is three-dimensional motion analysis (3DMA) using an optical system, which is useful in clinical settings because it can objectively provide both kinematic and kinetic parameter information [6]. However, these systems are installed in only a limited number of facilities and require specialized knowledge and time to operate and measure, making their frequent use difficult [7,8]. In addition, patients who are candidates for gait analysis have gait disorders such as decreased walking speed, endurance, and walking independence, and their gait disorder severity is higher than that of healthy individuals [9,10]. Therefore, with the existing gait analysis methods used in clinical settings, there is a high probability that the presence of an assistant during gait analysis to prevent a patient from falling will lead to restrictions in the placement and setting of the measurement equipment [11,12,13].

In the field of clinical gait analysis, there is a clear need for a simple and quantitative evaluation method that can be used for patients with a wide range of gait severities [4]. Several reports in recent years have described markerless motion analysis using RGB-D cameras that are capable of acquiring both color and depth images [14,15,16]. In previous studies, the advantages of using RGB-D cameras for gait analyses were described as the elimination of errors caused by the participant’s skin movement, marker positioning, and ease of measurement using posture estimation technology, which does not rely on reflective markers attached to the participant’s skin [15,16]. Previous studies of markerless gait analysis using multiple RGB-D cameras in healthy adults have demonstrated the possibility of measuring spatiotemporal and kinematic parameters with the same accuracy as that of 3DMA [17,18]. Moreover, a previous study of markerless motion analysis using a single RGB-D camera in stroke patients reported moderate accuracy for spatiotemporal parameters (walking speed, stance time, swing time, and step length) and kinematic parameters (hip and knee joint angles). Additionally, the findings demonstrated the validity of this approach in conjunction with clinical assessments [19]. Recently, the accuracy of gait analysis using a single RGB-D camera has been reported, assuming that it could be used in clinical settings [20,21]. Although the verification was conducted in a limited walking environment or on a treadmill, it has been reported that it is possible to measure spatiotemporal parameters with the same accuracy as a conventional optical system for 3DMA, even with a single camera [22,23]. Therefore, markerless gait analysis using an RGB-D camera is anticipated to become a new method for gait analysis in clinical settings.

However, when considering the use of this technique in clinical settings, and enabling the measurement of patients with a wide range of walking severities, it is essential to consider the effects of the presence of a walking assistant. However, there are currently no reports that consider or verify this. By considering the presence of a walking assistant, we verified the reliability and consistency of the time factors during walking, which were the focus of gait analysis. It is feasible to perform gait analysis with high reliability and measurement accuracy, even in clinical settings where a walking assistant is present. Furthermore, it is possible to establish a new gait analysis method with high reliability in clinical settings.

This study aimed to compare the intraday reliability of spatiotemporal parameters obtained from markerless gait analysis using an RGB-D camera with and without a simulated walking assistant and compare the consistency with a 3DMA system using an optical system with and without a simulated walking assistant. We hypothesized that markerless gait analysis using an RGB-D camera has intraday reliability because it can eliminate errors caused by the movement of the participant’s skin and marker placement compared with a 3DMA system using an optical system, which can eliminate the possibility that the participants’ markers are covered by an assistant during gait evaluation. Moreover, it is possible to measure the same data with or without a walking assistant, assuming that the data obtained from the 3DMA system using an optical system are true values.

## 2. Materials and Methods

### 2.1. Participants

The participants were recruited through the distribution of posters and utilization of the opportunity method. The participants were 20 healthy adults (mean age, 23.85 years; mean height, 164.54 cm; mean weight, 53.05 kg; 10 men and 10 women) who had no underlying neurological or orthopedic disorders that could affect their walking ability. This study was conducted in accordance with the ethical standards of the Ritsumeikan University Ethics Committee for Medical and Health Research Involving Human Subjects (approval no. BKC-LSMH-2022-004) and the Ethics Committee of Ukai Rehabilitation Hospital (approval no. 4-0005). All participants provided written informed consent in accordance with the Declaration of Helsinki.

### 2.2. Experimental Setup and Protocol

**Figure 1:**
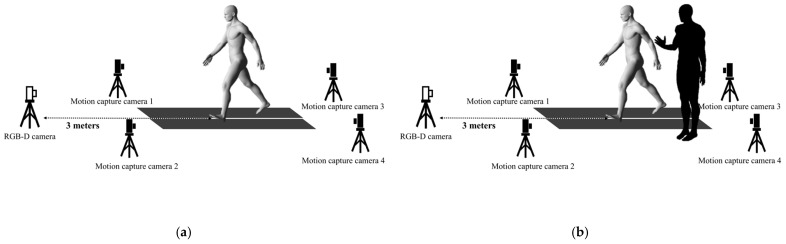
Setup adopted for the data acquisition process. The two panels show a sketch of the setup including the position of the four infrared (black) and one RGB (white) cameras. (**a**) This setup was adopted for data acquisition when the participant walked without assistance. (**b**) This setup was adopted for data acquisition when the participant walked with assistance. The assistant was positioned to the participant’s left.

The data were collected from a rehabilitation room at Ukai Rehabilitation Hospital (Keizan Medical Corporation). Measurements were obtained using a low-floor dual treadmill (DLF-55; OHTAKE, Iwate, Japan). The positions of the cameras used for data capture are shown in Figure 1. Before the measurements were taken, it was ascertained that the markers could be captured in situ using a minimum of three cameras with and without a walking assistant. The measurement environment was constructed using an optical motion capture system comprising four capture cameras (KISSEI COMTEC, Nagano, Japan), and the marker coordinate data attached to the body were acquired. During the measurements, the participants were instructed to wear blue clothing. For the lower body, shorts were used to ensure that the knee joints were visible. To avoid interference with the measurement process, the participants carried no bags, accessories, or additional items during the trial. Reflective markers were attached using double-sided tape to 18 points on the left and right sides of the body (acromion, anterior superior iliac spine, posterior superior iliac spine, greater trochanter, lateral epicondyle of the femur, medial epicondyle of the femur, lateral malleolus, medial malleolus, and metatarsal head of the fifth toe). Moreover, an RGB-D camera (RealSense D435i; Intel, Santa Clara, CA, USA) was used in conjunction with an Intel skeleton-tracking SDK. This SDK is compatible with RGB-D cameras and can estimate the skeletons of 12 joints. Python software (version 3.7) was used to estimate the central positions of the hip, knee, and ankle joints [24]. The camera was set up 3 m in front of each participant in the direction of travel according to the measurement conditions in a previous study [23]; the measurements were then taken. Walking speed during motion measurement was set at 2.9 km/h. The participant first walked without a simulated walking assistant; subsequently, walking was measured with a simulated walking assistant at the same treadmill speed. The assistant’s position while walking was set at the side, the most common position in actual clinical settings. The assistant was positioned to the left of the participant to assist them immediately (Figure 1). The number of participants was measured again after 72 h. During the second measurement, meticulous attention was paid to maintaining the camera setup and the assistants’ positions while walking. In addition, during the initial measurements and retests, the participants wore identically fitted blue clothing and shorts to ensure measurement condition consistency.

### 2.3. Data Processing and Estimation of Gait Parameters

The data obtained using the RealSense D435i (Intel, Santa Clara, CA, USA) were acquired at 30 Hz using the skeleton-tracking SDK application. The data obtained using the 3DMA were also acquired at 30 Hz. The camera coordinates were matched in advance using a calibration frame for each camera. The 3DMA gait analysis extracts the marker coordinate data attached to the body and processes the data using a low-pass Butterworth filter at 10 Hz. To estimate the gait cycle, we used the center of the ankle joint to estimate heel contact and toe-off as reported in previous studies [25,26]. We identified the gait cycle and classified the stance and swing phases to calculate step length [25].

The RGB-D camera gait analysis was performed using a dedicated Python script with the hip, knee, and ankle joint center position coordinates collected from the SDK. The collected data were processed using the same filtering process as that used for the 3DMA. Similar to the 3DMA, heel contact and toe-off were estimated from the ankle joint center position coordinates, the gait cycle was identified, and the stance and swing phases were classified to calculate the stride length [25]. For the 3DMA and RGB-D cameras, the center positions of the hip, knee, and ankle joints were calculated as described previously [27], while the analysis was conducted over five walking cycles from which the walking coordinate data of the target could be stably extracted. In this study, as an initial step in verifying the reliability and consistency of markerless gait analysis methods with and without a walking assistant, we analyzed spatiotemporal parameters (stride length, stance time, swing time, and gait cycle time) that are widely used in clinical settings [28]. We also incorporated lower-limb joint center position coordinates as foundational data for multidimensional analyses such as kinematics. This selection was made to establish a solid basis for future studies that aim to include more detailed metrics such as joint angles, angular velocities, and symmetry indices.

### 2.4. Statistical Analysis

The obtained spatiotemporal parameters (stride length, stance time, swing time, and gait cycle time) were used for the statistical analysis. First, we used the Shapiro–Wilk test to assess data normality. A correlation analysis was conducted to evaluate the relationship between participant characteristics (sex, height, and weight) and gait parameters. After normality was confirmed, the intraday reliability of the spatiotemporal parameters obtained from the gait analysis using the 3DMA and RGB-D cameras with and without a simulated walking assistant was verified using the intraclass correlation coefficient (ICC [1]), and the results were compared between measurement devices. Next, assuming that the data obtained from the 3DMA were true values, we used the ICC (2, 1) to evaluate the degree of consistency between the true value and the parameters obtained from the walking analysis using the RGB-D camera and compared them between conditions with and without a walking assistant. We also used the ICC (2, 1) to verify the consistency of the walking analysis using an RGB-D camera with and without a simulated walking assistant. Finally, the ICC (3, k) was used to assess the reproducibility of measurements across the measurement devices and under conditions with and without a walking assistant. The ICCs were classified into the following categories: <0.5  =  poor reliability; 0.5–0.75  =  moderate reliability; 0.75–0.9  =  good reliability; and >0.9  =  excellent reliability [29].

Furthermore, to evaluate the consistency of the spatiotemporal parameters obtained from the gait analysis of each measurement device, we used Bland–Altman analysis to check for systematic errors [30]. We also conducted the same analysis on the RGB-D camera measurements with and without a walking assistant during the retest to assess how the presence of a walking assistant affected the measurements. In systematic bias, fixed bias can be considered absent if a 95% confidence interval of the difference between two measurements is included [30]. Furthermore, the proportional bias can be determined by testing the difference between two measurements and the correlation between two average data groups [31]. When the absence of systematic bias was confirmed, the measurement error was calculated for each group using the minimum detectable change (MDC) since the difference between multiple measurements can be limited to random errors. MDC indicates the marginal range in which the change between two measurements obtained by repeated measurements, such as retests, is due to measurement errors. MDC_95_, a 95% confidence interval of the MDC, is generally used [32]. MDC_95_ is calculated using the following Equation (1):

*MDC*_95_ = *SEM* × 1.96 × √2 (1)

Although several methods for calculating the standard error of measurement (SEM) included in the MDC formula have been reported [31,32,33], the differences between the SEM calculated using these methods and the respective MDC values calculated using the SEM values are reportedly negligible, and the differences are negligible in clinical applications. In this study, SEM was used as previously reported [34]. Using the above formula, the MDC values for each parameter between the measuring devices were calculated with and without assistance while walking and the results were compared. We also calculated the MDC of the RGB-D camera measurements with and without walking assistance during the retest and evaluated the reproducibility under these conditions. Finally, the coefficient of multiple correlation (CMC) was used to verify the similarity of the time-series waveforms of the center position coordinates of each lower-limb joint during the gait cycle for each measurement device [35]. Statistical analyses were performed using SPSS (version 28.0), and the significance level was set at 5%.

## 3. Results

The results of the Shapiro–Wilk test showed that the spatiotemporal parameters (stride length, stance time, swing time, and gait cycle time) obtained from the gait analysis using each measurement device were normal. Table 1 summarizes the gait analysis data. A correlation analysis revealed no significant differences in gait parameters based on sex, height, or weight (all *p* > 0.05). This finding suggests that the measured parameters were consistent across the participants’ characteristics within the healthy population.

The ICC (1, 1) results with versus without the simulated walking assistant are listed in Table 2. The reliability of the gait analysis of each measurement device, with or without an assistant, was high as a result of the ICC (1, 1), with a range of 0.77–0.85, and no differences were noted between measurement devices or with versus without a walking assistant.

Next, assuming that the data obtained from the 3DMA were true values, Table 3 shows the results of ICC (2, 1) with versus without a simulated walking assistant to evaluate the degree of consistency between the true values and the parameters of the gait analysis obtained using the RGB-D camera. As a result of ICC (2, 1), the walking analysis of each measurement device had moderate consistency in the range of 0.70–0.74, and there was no significant change in the ICC (2, 1) value with or without a walking assistant.

The results of the walking analysis using the RGB-D camera with and without assistance during walking are listed in Table 4. The ICC (2, 1) results showed a high level of consistency in the range of 0.86–0.89.

The results of the reproducibility of the measurements with and without a walking assistant are listed in Table 5. The reproducibility of the gait analysis of each measurement device, with or without an assistant, was high as a result of the ICC (3, k), with a range of 0.76–0.79, and no differences were found between measurement devices or with versus without a walking assistant.

The Bland–Altman analysis showed no fixed or proportional bias between measuring devices. The MDC showed the following error values between the measurement devices used in the gait analysis: step length (without assistance, 3.37 cm; with assistance, 3.56 cm); stance phase time (without assistance, 0.18 s; with assistance, 0.19 s); swing phase time (without assistance, 0.12 s; with assistance, 0.11 s); and gait cycle time (without assistance, 0.16 s; with assistance, 0.17 s). Moreover, there were no differences in MDC between the measurement devices with versus without assistance (Table 6 and Table 7).

In addition, the error values when utilizing an RGB-D camera for gait analysis during a retest with versus without a walking assistant were as follows: step length, 2.24 cm vs. 2.44 cm; stance phase time, 0.12 s vs. 0.13 s; swing phase time, 0.09 s vs. 0.10 s; and gait cycle time, 0.13 s vs. 0.13 s. The analysis revealed no statistically significant differences in the MDC between the conditions with or without assistance (Table 8 and Table 9).

Finally, Table 10 presents the results of the CMC, which indicated the similarity of the time-series waveform of the lower-limb joint center position coordinates in the gait analysis between the two measurement devices. The CMC was 0.79–0.91 without an assistant and 0.81–0.89 with an assistant, and there was no change depending on assistant status. However, the CMC tended to decrease toward the distal lower limbs.

## 4. Discussion

This study aimed to evaluate the intraday reliability of spatiotemporal parameters obtained from markerless gait analysis using an RGB-D camera with versus without a simulated assistant during walking and compare it with the consistency of the same parameters obtained from a 3DMA system using an optical system with versus without a simulated assistant during walking. The results showed that the markerless gait analysis using an RGB-D camera has intraday reliability regardless of whether an assistant is present during walking and that it is possible to measure equally well with or without a walking assistant, assuming that the data obtained from a 3DMA system using an optical system are the true value. To the best of our knowledge, this is the first study to examine the reliability and consistency of gait analysis measurements using an RGB-D camera while considering the effects of the presence of an assistant during walking. The findings of this study are expected to contribute to the development of new gait analysis methods for clinical settings.

The present study adopted treadmill gait analysis as the experimental setup. Treadmills are staples used in clinical settings and research owing to their ability to provide controlled walking conditions and ensure patient safety. Specifically, treadmills are advantageous for patients with walking disorders resulting from neurological and orthopedic conditions, including stroke [36], cerebral palsy [37], Parkinson’s disease [38], and knee osteoarthritis [39]. Furthermore, treadmills enable uninterrupted data collection while maintaining a consistent walking speed, thereby facilitating the development of reliable gait analysis methods [40]. However, while some studies have reported similarities between treadmill and ground walking in terms of spatiotemporal, kinematic, kinetic, electromyographic, and energy consumption parameters [41], others have identified significant differences [42,43]. One potential factor contributing to these discrepancies is the difference in comfortable walking speeds between treadmill and overground walking [44]. In this study, treadmill speed was predetermined for all participants, which may have influenced the outcomes. These points should be considered when interpreting the results and applying treadmill-based gait analysis methods in clinical settings.

In this study, the ICC (1, 1) and ICC (3, k) for each measurement device in the gait analysis was ≥0.7 regardless of whether an assistant was present during walking. As previous studies have reported that an ICC ≥ 0.7 indicates good reliability [29], it was clear that markerless gait analysis using an RGB-D camera had good intraday reliability and inter-session reproducibility regardless of whether an assistant was present during walking. In a previous study of gait analysis using a single RGB-D camera in a treadmill setup similar to that used herein [23], the ICC (1, 1) was similar. One problem with 3DMA reliability and reproducibility is that errors can occur owing to participant skin movement and marker placement [45]. However, in markerless gait analysis using an RGB-D camera, these effects were eliminated; this study also had high reliability. Furthermore, in this verification, the inter-session reproducibility was high regardless of whether a walking assistant was present, and the error values were close to those reported in previous studies [46].

Regarding the time factors during walking that are often used in clinical settings, the measurement errors that occur between markerless gait analysis using RGB-D cameras and 3DMA measurement devices with or without an assistant during walking were approximately 0.12–0.18 s for the gait cycle and the stance and swing times within the gait cycle, and the step length was approximately 3.37–3.56 cm. In previous studies, the MDC for the 3MDA spatiotemporal factor was reportedly in the range of 0.02–0.08 s for the time factor and 3 cm for the step length [47], and the MDC obtained in this verification was about 0.1 s for the time factor and had a larger error value than that of the previous study. In markerless gait analysis using an RGB-D camera, the difficulty identifying foot landmarks owing to footwear, clothing, and the laboratory environment [15], as well as the sampling rate of the RGB-D camera used herein (30 Hz), may have resulted in slightly larger error values compared with the 3DMA method. However, a previous study reported that the MDC of the 10-m walking test in patients with stroke was 1.58–5.25 s [48] and the error in the time factor during walking that occurs in markerless gait analysis using an RGB-D camera is minimal and considered acceptable in clinical settings.

Based on the results of this study, markerless gait analysis using an RGB-D camera enabled measurements comparable to those obtained using 3DMA, assuming data from the latter as the standard regardless of the presence of assistance during walking. In this study, the measurement environment was on a treadmill, and the assistant was in a position in which they did not move even when the participant was walking; therefore, we believe that it would be possible to accurately measure even with an assistant in an environment similar to that used in this study. In clinical settings, patients who participate in gait analyses have a wide range of gait disorder severities [9,10]; in many cases, gait rehabilitation and assessments are performed with assistance. Therefore, the findings of this study are significant because gait analysis can be safely and accurately performed in patients with a wide range of gait disorder severities.

In markerless gait analyses, factors such as the participant’s clothing and camera setup are critical for ensuring accuracy. Previous studies have demonstrated that loose or baggy clothing can interfere with the estimation of joint position coordinates, while clothing color may affect the performance of infrared-based cameras [49]. To minimize these effects, the participants in this study wore fitted clothing in colors optimized for infrared reflection. Additionally, the RGB-D camera (RealSense D435i; Intel) used herein incorporates machine learning–based posture estimation, which reportedly reduces the influence of clothing on measurement accuracy [50]. Consequently, the effect of clothing on the data collected in this study was minimal. Moreover, identical clothing was used for the initial measurements and retests to ensure reproducibility. This approach minimizes the variability due to clothing and improves measurement reliability. However, it should be noted that this level of control may not be feasible in clinical settings where patients typically wear diverse types of clothing, including hospital gowns, everyday attire, or accessories (e.g., bags or medical devices). Such variations could influence markerless gait analysis accuracy and should be considered in future studies. Future studies should address these challenges by developing calibration methods or algorithms that can be adapted despite different clothing types [51,52]. In addition, standardizing clothing guidelines for clinical evaluations could further enhance their reliability [49]. Moreover, external factors such as lighting conditions, background complexity, and the presence of accessories should be investigated to improve the robustness and practicality of markerless gait analysis systems in clinical settings [15].

Regarding the camera setup, this study demonstrates that a single RGB-D camera positioned 3 m in front of each participant can facilitate reliable and consistent gait analysis measurements. This finding suggests that accurate and practical gait analyses can be achieved in clinical settings even with a single-camera setup. Previous studies have also reported that spatiotemporal parameters measured from the frontal camera position demonstrate high reliability [26,53]. However, the position and distance of the camera can influence measurement accuracy [54], and resolution loss becomes significant as the distance between the camera and participant increases [55]. Although this study verifies the reliability and consistency of a single RGB-D camera setup, it is plausible that the measurement accuracy could be further improved by employing multiple RGB-D cameras to minimize blind spots and expand the field of view [17,18]. Therefore, careful consideration of camera positioning and setup is essential for optimizing markerless gait analyses.

This study has some limitations. First, it should be noted that the participants were healthy adults. To assess the applicability of the method in clinical settings, it is necessary to include patients with diseases or disabilities or have participants simulate specific gait patterns. Addressing these limitations in future research is crucial for validating the clinical utility of this markerless gait analysis. Second, the gait analysis parameters calculated herein included only spatiotemporal factors and did not cover all parameters used in clinical gait analyses. Third, this study examined only treadmill walking. In the next stage, it will be necessary to compare treadmill and overground walking at different speeds to clarify its usefulness in clinical settings. By verifying these factors, the clinical usefulness of markerless gait analysis using RGB cameras can be adopted.

## 5. Conclusions

This study’s findings suggest that RGB-D cameras can reliably and consistently capture gait parameters even with walking assistance, thereby offering a feasible solution for gait analysis in clinical environments in which traditional motion capture may be challenging.

However, the applicability of this method to clinical settings requires further validation, particularly in patients with gait disorders. Future studies should evaluate its accuracy and reliability in patients with abnormal gait patterns and investigate optimal measurement conditions, including the influence of clothing, camera placement, and overground walking conditions.

Further research is essential to establish the robustness and practicality of markerless gait analysis using RGB-D cameras, although this study highlights its potential for clinical settings.

## Figures and Tables

**Table 1:** Gait parameter analysis results without versus with assistance.

Gait Parameter	Without Assistance				With Assistance			
	3DMA		RGB-D		3DMA		RGB-D	
	Session 1	Session 2	Session 1	Session 2	Session 1	Session 2	Session 1	Session 2
Stride length (cm)	51.15 ± 4.07	49.18 ± 3.25	48.52 ± 2.23	50.56 ± 2.12	51.28 ± 3.84	51.42 ± 3.01	49.87 ± 2.14	52.39 ± 3.04
Stance time (s)	0.73 ± 0.04	0.74 ± 0.05	0.72 ± 0.05	0.73 ± 0.05	0.73 ± 0.03	0.74 ± 0.04	0.72 ± 0.06	0.72 ± 0.03
Swing time (s)	0.53 ± 0.04	0.52 ± 0.08	0.56 ± 0.04	0.55 ± 0.03	0.52 ± 0.03	0.53 ± 0.05	0.56 ± 0.03	0.54 ± 0.03
Gait cycle time (s)	1.26 ± 0.06	1.27 ± 0.08	1.28 ± 0.08	1.28 ± 0.07	1.26 ± 0.08	1.27 ± 0.06	1.30 ± 0.08	1.27 ± 0.05

Data are expressed as the mean ± standard deviation; 3DMA, three-dimensional motion analysis, RGB-D, color and depth camera.

**Table 2:** Reliability of gait analysis for each measurement device.

Gait Parameter	3DMA				RGB-D Camera			
	Without Assistance		With Assistance		Without Assistance		With Assistance	
	ICC (1, 1)	95% Confidence Interval	ICC (1, 1)	95% Confidence Interval	ICC (1, 1)	95% Confidence Interval	ICC (1, 1)	95% Confidence Interval
Stride length (cm)	0.83	0.57–0.94	0.83	0.55–0.92	0.79	0.54–0.91	0.78	0.57–0.91
Stance time (s)	0.85	0.65–0.91	0.84	0.65–0.89	0.78	0.55–0.86	0.78	0.55–0.89
Swing time (s)	0.86	0.69–0.93	0.83	0.64–0.88	0.77	0.58–0.85	0.78	0.56–0.87
Gait cycle time (s)	0.85	0.71–0.93	0.82	0.72–0.93	0.76	0.55–0.84	0.77	0.54–0.85

3DMA, three-dimensional motion analysis; ICC, intraclass correlation coefficient; RGB-D, color and depth camera.

**Table 3:** Intraclass correlation coefficient (2, 1) between devices in gait analysis.

Gait Parameter	Without Assistance		With Assistance	
	ICC (2, 1)	95% Confidence Interval	ICC (2, 1)	95% Confidence Interval
Stride length (cm)	0.71	0.52–0.84	0.72	0.51–0.86
Stance time (s)	0.74	0.51–0.87	0.71	0.56–0.84
Swing time (s)	0.72	0.54–0.87	0.72	0.54–0.85
Gait cycle time (s)	0.70	0.51–0.83	0.71	0.52–0.81

ICC, intraclass correlation coefficient.

**Table 4:** Intraclass correlation coefficient (2, 1) in gait analysis with versus without assistance using an RGB-D camera.

Gait Parameter	ICC (2, 1)	95% Confidence Interval
Stride length (cm)	0.86	0.59–0.94
Stance time (s)	0.88	0.56–0.87
Swing time (s)	0.89	0.59–0.88
Gait cycle time (s)	0.88	0.51–0.87

**Table 5:** Reproducibility of gait analysis for each measurement device.

Gait Parameter	3DMA				RGB-D Camera			
	Without Assistance		With Assistance		Without Assistance		With Assistance	
	ICC (3, k)	95% Confidence Interval	ICC (3, k)	95% Confidence Interval	ICC (3, k)	95% Confidence Interval	ICC (3, k)	95% Confidence Interval
Stride length (cm)	0.78	0.58–0.87	0.77	0.57–0.84	0.78	0.57–0.91	0.77	0.54–0.91
Stance time (s)	0.78	0.51–0.91	0.77	0.55–0.86	0.78	0.55–0.89	0.78	0.55–0.86
Swing time (s)	0.78	0.61–0.92	0.79	0.55–0.84	0.78	0.56–0.87	0.77	0.58–0.85
Gait cycle time (s)	0.77	0.55–0.88	0.78	0.54–0.85	0.77	0.54–0.85	0.76	0.55–0.84

3DMA, three-dimensional motion analysis; ICC, intraclass correlation coefficient; RGB-D camera, color and depth camera.

**Table 6:** Bland–Altman analysis of each gait analysis and error values between gait analyses without assistance.

Gait Parameter	Bland–Altman Analysis			SEM	MDC
	Fixed Bias	Proportional Bias			
	95% Confidence Interval	Correlation Coefficient	*p*-Value		
Stride length (cm)	−1.18 to 1.25	1.37	*p* > 0.05	1.22	3.37
Stance time (s)	−0.04 to 0.08	1.01	*p* > 0.05	0.06	0.18
Swing time (s)	−0.02 to 0.09	0.96	*p* > 0.05	0.04	0.12
Gait cycle time (s)	−0.08 to 0.05	1.01	*p* > 0.05	0.06	0.16

MDC, minimum detectable change; SEM, standard error of measurement.

**Table 7:** Bland–Altman analysis of each gait analysis and error values between gait analysis with assistance.

Gait Parameter	Bland–Altman Analysis			SEM	MDC
	Fixed Bias	Proportional Bias			
	95% Confidence Interval	Correlation Coefficient	*p*-Value		
Stride length (cm)	−0.67 to 1.12	1.22	*p* > 0.05	1.28	3.56
Stance time (s)	−0.03 to 0.08	1.08	*p* > 0.05	0.07	0.19
Swing time (s)	−0.02 to 0.08	0.99	*p* > 0.05	0.04	0.11
Gait cycle time (s)	−0.05 to 0.07	1.02	*p* > 0.05	0.06	0.17

MDC, minimum detectable change; SEM, standard error of measurement.

**Table 8:** Bland–Altman analysis of gait analysis using RGB-D camera without assistance.

Gait Parameter	Bland–Altman Analysis			SEM	MDC
	Fixed Bias	Proportional Bias			
	95% Confidence Interval	Correlation Coefficient	*p*-Value		
Stride length (cm)	−1.00 to 1.13	1.35	*p* > 0.05	0.80	2.24
Stance time (s)	−0.09 to 0.18	1.22	*p* > 0.05	0.04	0.12
Swing time (s)	−0.10 to 0.05	0.88	*p* > 0.05	0.03	0.09
Gait cycle time (s)	−0.08 to 0.14	1.12	*p* > 0.05	0.05	0.13

MDC, minimum detectable change; SEM, standard error of measurement.

**Table 9:** Bland–Altman analysis of gait analysis using RGB-D camera with assistance.

Gait Parameter	Bland–Altman Analysis			SEM	MDC
	Fixed Bias	Proportional Bias			
	95% Confidence Interval	Correlation Coefficient	*p*-Value		
Stride length (cm)	−1.57 to 0.72	1.11	*p* > 0.05	0.88	2.44
Stance time (s)	−0.03 to 0.24	1.04	*p* > 0.05	0.05	0.13
Swing time (s)	−0.04 to 0.06	0.99	*p* > 0.05	0.04	0.10
Gait cycle time (s)	−0.04 to 0.15	1.06	*p* > 0.05	0.05	0.13

MDC, minimum detectable change; SEM, standard error of measurement.

**Table 10:** Coefficient of multiple correlation between devices in gait analysis.

Gait Parameter	Without Assistance			With Assistance		
	Coefficient of Multiple Correlation			Coefficient of Multiple Correlation		
	x–y	y–z	x–z	x–y	y–z	x–z
Hip joint central position	0.88 ± 0.13	0.91 ± 0.19	0.87 ± 0.11	0.89 ± 0.12	0.89 ± 0.13	0.88 ± 0.15
Knee joint central position	0.82 ± 0.22	0.86 ± 0.20	0.86 ± 0.23	0.81 ± 0.24	0.87 ± 0.17	0.85 ± 0.29
Ankle joint central position	0.79 ± 0.28	0.80 ± 0.22	0.84 ± 0.20	0.81 ± 0.25	0.81 ± 0.24	0.84 ± 0.21

## Data Availability

The data supporting the findings of this study are available upon reasonable request. Owing to ethical considerations and licensing restrictions, only anonymized and summarized datasets can be shared. Researchers interested in accessing the data should contact the corresponding author and provide a clear outline of the intended use. Permission to access the data will be granted according to ethical guidelines and institutional regulations. In future studies, we aim to make the datasets more openly accessible by implementing advanced anonymization techniques and obtaining broader participant consent.
